# Disparate thermostability profiles and HN gene domains of field isolates of Newcastle disease virus from live bird markets and waterfowl in Uganda

**DOI:** 10.1186/s12985-016-0560-0

**Published:** 2016-06-21

**Authors:** John Bosco Omony, Agnes Wanyana, Kizito K. Mugimba, Halid Kirunda, Jessica Lukanga Nakavuma, Maxwell Otim-Onapa, Denis Karuhize Byarugaba

**Affiliations:** College of Veterinary Medicine, Makerere University, P.O. Box 7062, Kampala, Uganda; Uganda Industrial Research Institute (UIRI), P.O. Box 7086, Kampala, Uganda; Uganda National Council of Science & Technology (UNCST), P.O. Box 6884, Kampala, Uganda; Mbarara Zonal Agricultural Research and Development Institute (MBAZARDI), National Agricultural Research Organisation (NARO), P.O. Box 389,, Mbarara, Uganda

**Keywords:** Thermostability, Infectivity, Thermostable isolate, Hemagglutionation assay, HN gene

## Abstract

**Background:**

Uganda poultry production is still faced with frequent outbreaks of Newcastle disease (ND) in the backyard free-range systems despite the accessibility of cross protective vaccines. Live bird markets and waterfowl has long been reported as a major source of disease spread as well as potential sources of avirulent strains that may mutate to virulent strains. ND-virus has been reported enzootic in Ugandan poultry but limited studies have been conducted to ascertain thermostability phenotypes of the Ugandan ND-virus strains and to understand how these relate to vaccine strains.

**Methods:**

This study evaluated thermostability of 168 ND-virus field isolates recovered from live bird markets and waterfowls in Uganda compared to two live commercial vaccine strains (I_2_ and LaSota) by standard thermostability procedures and Hemagglutinin-Neuraminidase (HN) gene domains. The known pathotypes with thermostability profiles were compared at HN amino acid sequences.

**Results:**

Field isolates displayed disparate heat stability and HN gene domains. Thermolabile isolates were inactivated within 15 min, while the most thermostable isolates were inactivated in 120 min. Four thermostable isolates had more than 2 log_2_ heamaglutinin (HA) titers during heat treatment and the infectivity of 9.8 geometric mean of log_10_ EID_50 %_ in embryonated eggs. One isolate from this study exhibited a comparable thermostability and stable infectivity titers after serial passages, to that of reference commercial vaccine was recommended for immunogenicity and protection studies.

**Conclusion:**

The occurrence of ND-virus strains in waterfowl and live bird markets with disparate thermostability and varying HN gene domains indicate circulation of different thermostable and thermolabile ND-virus pathotypes in the country.

## Background

Newcastle disease (ND) is a contagious poultry infection that usually invokes trade barriers and exerting great economic threats [1]. Newcastle disease virus (ND-virus) genome contains 6 genes encoding for structural proteins positioned from 3′ to 5′: Nucleoprotein (NP), Phosphoprotein (P), Matrix (M), Fusion (F) Hemagglutinin-Neuraminidase (HN) and polymerase (L) [23]. The physico-chemical properties of these proteins have been extensively used to categorize strains. According to World Organization for Animal Health [27], for a ND-virus to be notified as pathogenic, it has to meet the criteria of Intracerebral pathogenicity Index (ICPI) of beyond 0.7. Today, field strains are categorized by virulence into five pathotypes [3, 5, 6], confirmed by multiple basic amino acid sequences in the fusion (F) protein cleavage site (FPCS) [12, 26].

Great importance is attached to the thermostability of live vaccinates because of their retained potency in areas with poor transport, storage facilities, human error and power shortages. Studies in search of new thermostable isolates assess virulence of pathotypes by determining the amino acid sequences at the F protein cleavage site (FPCS), mean death time (MDT) and Intracerebral pathogenicity indices (ICPI). In areas where there is frequent use of vaccines, selective pressure leading to new strains or failed vaccination has been recorded due to the use of phylogenetically divergent vaccine strains from the circulating local ND-viruses [24, 28]. Besides, ND-virus isolates with amino acids at FPCS typical of low-virulent type and ICPI values typical of highly virulent strains have been reported [37]. Such places are experiencing continual outbreaks of velogenic ND in backyard and vaccinated flocks [1, 4].

HN gene is known to play crucial multifunctions in infectivity [14, 42]. It has also been confirmed through reverse genetics using cDNA clones (ICS) as a crucial determinant of NDV thermostability with conferred complete protection of birds [43]. Thermostability characterization of heat-stable ND-viruses relating to HN gene will improve understanding of the molecular basis of ND-virus thermostability. This will augment rational design of NDV vaccines to solve managerial problems in scavenging rural chicken [18] or further evaluate performance of established vaccine strains.

Recovery of virulent ND strains in birds vaccinated with avirulent vaccines strains or in ND-virus endemic countries raises concerns whether these strains are derived from indigenous or vaccine strains [2]. In Uganda, available ND vaccines used are namely: LaSota strain and I_2_ strain with well-defined genotypes. However, the recent ND-virus isolates from Ugandan live bird markets indicate a high pathogenicity and low evolution [8] with no thermostability phenotypes. Several studies have recovered low-virulent, thermostable ND-virus in several species of birds [5, 33]. Such strains have been evaluated as vaccine candidates to protect village birds in the tropics where temperatures are high and local farmers either lack or are unable to pay for the cold-chain needed to sustain live thermolabile ND-virus vaccine usage [35].

Thermostability of hemagglutinins of ND-virus is retained through many serial passages in embryonating eggs and can distinguish one culture from another [13]. The basis of thermostability testing of ND-virus is that all strains have hemagglutinin surface proteins, which agglutinates chicken RBCs in vitro, and Neuraminidase enzyme that promotes virus release from infected cells. The activity of these surface glyco-proteins is used to detect hemagglutinating viruses in the family [27], and to follow their stability when the virus is exposed at different temperatures [1]. Since the development of a thermostable Australian V4 ND vaccine strain, several thermostability testing methods have been used to evaluate live ND-virus isolates and vaccine candidates for ability to survive under different temperatures [19, 21, 22, 36].

The aim of the current study was to determine the thermostability of ND-virus isolates recovered from live bird markets (LBM) and waterfowls and relate this phenotype to HN gene sequence.

## Materials and methods

### ND-virus isolates

A total of 168 ND-virus isolates were used in the current study. These field ND-viruses were isolated in Uganda during 2011 from LBM comprised of local chickens, turkeys and ducks. The isolates from waterfowl were recovered from freshly voided fecal droppings of migratory and resident waterfowls in selected landing sites. Sixty seven percent (112/168) of the viruses of LBM were isolated from cloacal (C) and/oro-pharyngeal (P) swabs, while thirty three percent (56/168) of isolates from waterfowls were recovered from fresh fecal (F) matter. Initially, viruses were passaged twice in 10–day-old embryonated chicken eggs (ECE) obtained from ND-free flocks and clean isolates obtained in chicken embryonated fibroblasts (CEF). The allantoic fluid was aseptically harvested, virus presence confirmed by HI test according to standard procedures and analyzed further or stored at −20 °C as virus stocks. Biological characterization for chicken ND isolates were described elsewhere [8]. Two live commercial vaccines (I_2_ and LaSota) were used as controls throughout the study.

The presence of the ND-virus was confirmed by PCR and serologically with positive sera in HI test [8, 27]; using 0.5 % (v/v) washed ND-virus–free chicken RBCs according to standard procedures. Serum was generated in in-house rabbits immunized with ND-virus - LaSota vaccine strain (Laborotorios Hipra, S.A, Spain).

### Assessment of thermostability

HA and infectivity titers for all the stock viruses were determined by standard microtiter plate hemagglutionation assay [27] and the viability of selected isolates by end point infectivity assay [30]. Nine paired sealed vials containing 0.5 mL aliquots of each ND-virus isolate were thawed once. One pair was left on ice while the other eight were incubated on a water bath (Polypro Bath® CA, USA) kept at a constant temperature (56 ± 0.5 °C). At regular interval of 15, 30, 45, 60, 75, 90, 105 and 120 min, a pair of vials was removed and chilled quickly on ice to stop heat inactivation. All the aliquots were assayed for HA activity by standard methods [27, 30]. The isolates that showed HA titers at 56 °C after 2 h were further analyzed for regression analysis of HA activity using rate constants (*k*) and among these, avirulent isolates were passaged five times without heating intervals. Additionally, to determine the effect of heat on the viability or multiplication of the selected viruses, the vials incubated at 56 °C for 0, 30, and 60 min were assayed for virus viability and compared to the thermostable I_2_ strain. Briefly, 0.3 mL of the virus aliquot from each mentioned treatment was injected into three sets of eggs and incubated for 4 days before allantoic fluid was harvested, checked for the mean log_2_ HA titer and assayed for infectivity.

### Stability of infectivity

The infectivity stability of the selected avirulent viruses was evaluated and compared to vaccine strains (I_2_ and LaSota) in 10-day-old embryonated eggs. The infectivity evaluation of the isolates involved five passages. Briefly, tenfold serial dilutions were made in normal saline solution (Phosphate Buffered Saline, PBS) and five dilutions were selected. One hundred microliters (0.1 mL) of each of these dilutions: 10^-1^, 10^-6^, 10^-7^, 10^-8^ and 10^-9^ was inoculated in duplicate into five eggs, incubated for 4 days, and harvested for HA and median embryo infectivity dose at fifty percent (log_10_EID_50_) assays. This was carried out for five passages where both HA and infectivity assays were carried out for each passage. Additionally, the heamagglutionation units were measured following passages, to evaluate the replication competence of the isolates.

### Viral RNA extraction, RT-PCR and DNA sequencing

Viral RNA was extracted from all the HI positive allantoic fluid samples by using the QIAamp Viral RNA mini kit (Qiagen, USA) according to the manufacturer’s instructions and sequenced to obtain full HN sequences as described in [8].

### HN gene sequence analysis

To predict the HN relationship between the field ND-virus isolates and vaccine strains, multiple sequence alignment of HN gene of representative thermostable ND-virus isolates was done. Using the following accession numbers previously deposited in the Gene Bank for isolates; NDV/UG/MU/007- HG937536, NDV/UG/MU/010-HG937538, NDV/UG/MU/022- HG937542, NDV/UG/MU/039- HG937548, NDV/UG/MU/059-HG937553, NDV/UG/MU/098- HG937564 and NDV/UG/MU/111- HG937566. Together with other representative thermostable vaccines: TS09C- JX110635, I_2_ – AY935499, I_2_ progenitor – AY935500, NDV4-C - JX443519 and thermolabile: Lasota –JF950510, LaSota C5 – KC844235, and D58 strain- EU305607 and others were blast searched from NCBI GeneBank using the Bio-Edit software (North Carolina State University, USA) to generate sequence analysis data. The parameters used for the sequences analysis were multiple alignment (Clustal W), sequence identity plotter and sequence matrix at both amino acid and nucleotide levels. The B- cell epitope of HN gene was predicted in a computer algorithm using web server based on software www.bioinfo.tsinghua.edu.cn/epitope/EPMLR. The transmembrane domains were predicted using Dense Alignment Surface (DAS) transmembrane domain prediction server– http:www.sbc.su.se/-miklos/DAS/maindashtml [11].

### Statistical analyzes

Thermostability was analyzed by calculating the slope of the regression curve of HA activity. When appropriate, the data of exponential decline in activity of HA were presented as rate constants (k, expressed as mins^-1^), defined by first-order inactivation equation below as described [41].

$$ 2.303\  log10\ \left(V/Vo\right)=-kt $$

Isolates whose late time points, i.e., 105 or 120 min did not contain HA were excluded from analysis. The survival analysis for the isolates were titrated by standard median EID_50 %_ according to standard protocols [27]. All statistical analyzes were performed in GraphPad Prism, version 5.

## Results

### Thermostability of field ND-virus isolates at 56 °C

**Fig. 1:**
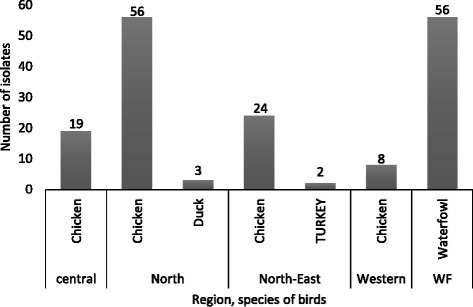
Distribution of ND-virus isolates by region and species of birds. The numbers above bars indicate isolates in different regions and bird species

The 168 field isolates showed HA titers greater than 16 log_2_ in embryonated eggs during the initial culture and were confirmed as ND-viruses by HI test and PCR. Regarding the source of poultry isolates, Northern region had the highest number of isolates compared to other regions. ND-viruses occurring in different species of poultry existing in the four regions of Uganda (Fig. 1). All the isolates used showed disparate thermostability at 56 °C ranging from 15 to 120 min (Table 1). Of these study isolates, 13.7 % (23/168) retained more than 2 log_2_ of their initial HA titers after 120 min and were regarded as thermostable while 86.3 % (145/168) without residual HA titer after the above time were considered thermolabile isolates. Of the 23 thermostable isolates, 13 were from LBM while 10 were from waterfowls (Table 1). Based on the heat inactivation rate constant (*k*) of HA activity, the thermostable strains were included in Table 2. When compared by rate constant of HA activity, 26.1 % (6/23) of the 23 thermostable ND-virus isolates were inactivated at a lower rate (higher heamagglutinin activity) than the reference I_2_ vaccine strain. Also all these isolates were from waterfowls.

**Table 1:** Thermostability profiles with numbers of NDV isolates by HA activity determined at 56 °C incubation

			Thermostability/inactivation time in min. at 56 °C								
REGIONS	District	Site	15	30	45	60	75	90	105	120	All
Central	KALUNGU	C	–	1	–	1	–	–	–	–	2
		P	–	1	–	2	1	–	–	–	4
	KIBOGA	C	–	1	–	–	–	–	–	–	1
		P	–	1	–	–	–	–	–	–	1
	LWENGO	C	2	–	–	–	–	–	–	–	2
		P	–	2	–	–	–	–	–	–	2
	MASAKA	C	–	1	–	–	–	–	–	1	2
		P	1	1	–	–	–	–	–	–	2
	MUKONO	P	1	–	–	–	–	–	–	–	1
	WAKISO	C	2	–	–	–	–	–	–	–	2
East	BUGIRI	C	–	1	1	–	–	–	–	–	2
		P	1	1	–	–	–	–	–	–	2
	IGANGA	C	–	1	1	–	–	–	–	–	2
		P	–	–	–	–	–	1	–	–	1
		T	–	2	–	–	1	–	–	–	3
	MAYUGE	P	–	1	–	–	–	–	–	–	1
	MBALE	C	1	–	–	–	–	–	–	1	2
		P	–	1	–	–	–	–	–	–	1
North	APAC	C	1	–	–	–	–	–	–	–	1
	ARUA	C	2	1	–	–	–	–	–	–	3
		P	3	–	1	–	–	–	–	–	4
	KOBOKO	C	–	2	–	–	–	–	–	–	2
		P	–	–	2	1	1	–	–	–	4
	KOLE	P	1	–	–	–	–	–	–	–	1
	LIRA	C	1	–	–	–	–	–	–	–	1
		P	1	–	–	–	–	–	–	–	1
	MARACH	C	1	1	–	–	–	–	1	–	3
		P	–	–	2	–	1	1	–	–	4
	OTUKE	C	–	–	1	–	–	–	–	–	1
	OYAM	C	5	1	–	–	–	1	–	2	9
		P	7	1	2	–	1	–	–	2	13
	YUMBE	C	1	–	–	–	–	–	–	–	1
		P	2	–	–	–	–	–	–	1	3
	ZOMBO	C	3	1	–	1	–	1	–	–	6
		P	–	–	1	–	–	–	–	–	1
North-East	ABIM	C	–	1	–	2	–	–	–	1	4
		P	–	1	–	–	–	–	–	1	2
	KOTIDO	C	–	1	–	–	–	–	–	–	1
		P	2	–	–	–	–	–	–	–	2
	KUMI	C	–	–	–	–	–	–	–	1	1
		P	–	–	1	1	2	–	–	–	4
	NAMUTUMBA	C	–	1	–	1	1	–	–	–	3
	SERERE	P	–	1	–	–	–	–	–	–	1
Western	BUNDIBUJO	C	–	–	1	–	–	–	–	1	2
	KABALE	P	–	1	–	–	–	–	–	–	1
	KABAROLE	C	1	–	–	–	–	–	–	–	1
	KASESE	F	1	–	–	–	1	–	1	2	5
		P	–	–	1	–	–	–	–	–	1
	KYENJOJO	P	–	1	–	–	–	–	–	–	1
	MBARARA	P	–	1	–	–	–	–	–	–	1
Waterfowl	JINJA	F	2	1	–	–	–	–	1	2	6
	RAKAI	F	10	1	1	–	–	–	–	3	15
	WAKISO	F	8	5	3	1	–	2	1	5	25
Number of Isolates			60	38	18	10	9	6	4	23	168

**Table 2:** Hemagglutination titers and heat inactivation rate constant (*k*) of thermostable ND-viruses incubated at 56 °C

	Arithmetic mean HA titer (log2) at various time intervals of heat inactivation (minutes)									Rate constant (k)
Virus Isolates	0	15	30	45	60	75	90	105	120	(10^3^ K/min^−1^)
NDV007/UG/MU/2011	5	5	5	5	5	5	4	4	4	2.23
NDV015/UG/MU/2011	6	6	6	6	5	5	4	4	4	4.26
NDV022/UG/MU/2011	8	8	8	8	8	8	8	8	3	4.36
NDV023/UG/MU/2011	7	7	6	5	5	4	4	3	1	12.62
NDV039/UG/MU/2011	8	8	8	8	7	7	6	5	4	5.44
NDV059/UG/MU/2011	8	8	7	7	6	6	5	4	3	7.59
NDV073/UG/MU/2011	5	5	5	5	4	4	3	2	1	11.59
NDV089/UG/MU/2011	3	3	3	3	3	3	2	1	1	9.45
NDV092/UG/MU/2011	5	5	4	3	2	2	2	1	1	14.51
NDV097/UG/MU/2011	8	4	4	4	4	4	4	4	4	3.08
NDV098/UG/MU/2011	5	4	4	3	2	2	2	2	2	8.38
NDV099/UG/MU/2011	5	5	4	4	4	3	3	3	1	9.82
NDV111/UG/MU/2011	6	5	4	4	3	2	2	2	2	10.25
NDV115/UG/MU/2011	6	6	6	6	6	6	6	6	4	1.80
NDV118/UG/MU/2011	7	7	7	7	7	7	7	7	7	0.00^b^
NDV119/UG/MU/2011	5	5	5	5	4	4	4	3	2	6.52
NDV133/UG/MU/2011	6	6	6	6	6	6	6	6	6	0.00^b^
NDV152/UG/MU/2011	6	6	6	6	6	6	6	6	6	0.00^b^
NDV158/UG/MU/2011	4	3	3	3	3	3	3	2	1	7.51
NDV173/UG/MU/2011	6	6	6	6	6	6	6	6	5	0.81^b^
NDV177/UG/MU/2011	6	6	6	6	6	6	6	5	5	1.22^b^
NDV178/UG/MU/2011	6	6	6	6	6	6	6	6	4	0.81^b^
NDV180/UG/MU/2011	6	6	6	5	5	4	4	3	3	6.54
*I*_*2*_ *vaccine*	11	10	10	10	10	10	10	9	9	1.24^a^
*LaSota vaccine*	8	≥1	–	–	–	–	–	–	–	245.97^a^

^a^Reference vaccines

^b^Most thermostable ND-virus, below I_2_ reference thermostable strain

**Fig. 2:**
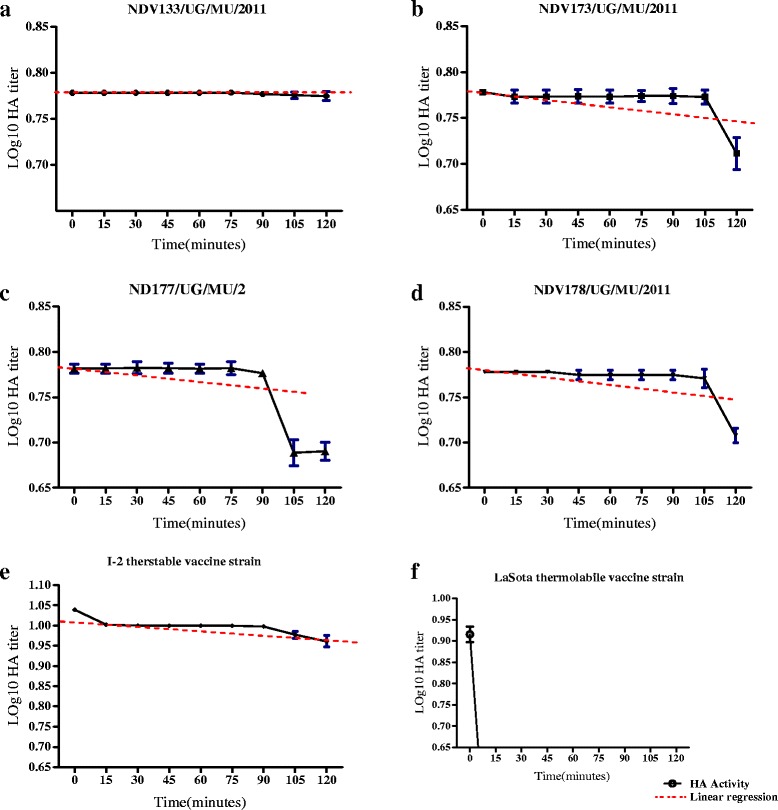
In vitro HA stability test of indicated thermostable field ND-virus at different time intervals. Incubation at 56 °C revealed loss of titer for isolates after 120 min (**a**–**d**). Thermostable I_2_ (**e**) and thermolabile LaSota (**f**) reference strains were included as controls. Values were averages of two independent tests (Mean ± SD, n = 2)

By plotting the logarithmically transformed (log_10_) HA of the selected avirulent isolates against time, the inactivation rates were further compared (Fig. 2). Except for NDV133/UG/MU/2011 and LaSota that yielded a monophasic curve (Fig. 2a and d), other selected thermostable isolates produced biphasic curves (Fig. 2b-d). For biphasic curves, from zero to 90 time points, a plateau that was observed was followed by a linear decline. Overall, isolate NDV133/UG/MU/2011 had better survival rate compared to NDV173/UG/MU/2011, NDV177/UG/MU/2011 and NDV178/UG/MU/2011 as avirulent thermostable isolates.

### Stability of infectivity upon passage

**Fig. 3:**
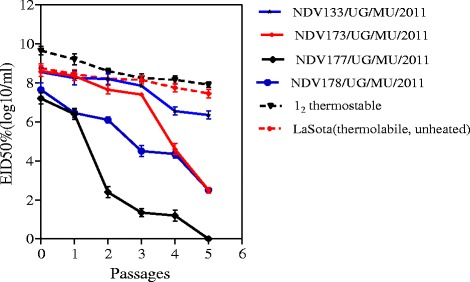
Stability of infectivity of indicated ND-virus strains by geometric mean log_10_EID_50%_ following serial passages. A greater change in the titers of field strains occurred at 4^th^ and 5^th^ passages compared to I_2_ reference vaccine. LaSota, a thermolabile vaccine was unheated initially but included to compare its viability with that of thermostable isolates. Values indicated are means of three independent tests (Mean ± SD, n = 3)

Four clean thermostable isolates were passaged five times and their infectivity shown in Fig. 3. Infectivity of the isolates decreased along the five serial passages. At the end of 3^rd^ passage, the infectivity of isolate NDV177/UG/MU/2011 rapidly declined to a geometric median log_10_ EID_50 %_/mL titer of 1.25, while that of NDV178/UG/MU/2011 was at 4.55 at the same passage. The infectivity titers of NDV133/UG/MU/2011 and NDV173/UG/MU/2011 isolates remained fairly close to that of I_2_ vaccine strain at geometric median log_10_ EID_50 %_ of 7.4. A greater change in infectivity of the isolates was at the fourth and fifth passages. Comparatively, one isolate NDV133/UG/MU/2011 retained more than 6.5 log_10_EID_50 %_/mL of its infectivity titer compared to I_2_ and LaSota of 8.05 log_10_EID_50 %_/mL and 7.5 log_10_EID_50 %_/mL respectively at the 5^th^ passage. The infectivity titers of isolates following a less than one hour time heat exposure were compared in Table 3. The ND-virus isolates NDV133/UG/MU/2011, NDV173/UG/MU/2011 and ND178/UG/MU/2011 retained more than 50 % of their log_10_EID_50 %_ infectivity within one hour of exposure at 56 °C_._ LaSota (thermolabile) vaccine strain had no detectable infectivity after 30 min of heat exposure while I_2_ (thermostable) strain retained its infectivity.

**Table 3:** HA and infectivity titers of avirulent, thermostable ND-viruses at 56 °C for 0, 30, 60 min

Virus	Parameter	Heat treatment time (minutes.) at 56 °C		
		0	30	60
NDV133/UG/MU/2011	^a^HA titer (log2)	2^6^	2^6^	2^6^
	^b^Infectivity titer	10^8.53^	10^8.35^	10^8.05^
NDV173/UG/MU/2011	^a^HA titer (log2)	2^6^	2^6^	2^6^
	^b^Infectivity titer	10^8.4^	10^8.25^	10^7.7^
NDV177/UG/MU/2011	^a^HA titer (log2)	2^6^	2^6^	2^6^
	^b^Infectivity titer	10^7.3^	10^6.3^	10^2.25^
NDV178/UG/MU/2011	^a^HA titer (log2)	2^6^	2^6^	2^6^
	^b^Infectivity titer	10^7.8^	10^6.45^	10^6.2^
I_2_ thermostable strain	^a^HA titer (log2)	2^11^	2^10^	2^10^
	^b^Infectivity titer	10^9.8^	10^9.5^	10^9.3^
LaSota thermolabile	^a^HA titer (log2)	2^9^	2^9^	2^9^
	^b^Infectivity titer	10^9.6^	–	–

^a^Hemagglutination assay (HA) titer presented as arithmetic mean HA log_2_ titer; and infectivity as log_10_

^b^50 % embryonated egg infectious dose (EID_50_/ml)

### HN gene sequences

The HN gene of Ugandan isolates contains coding sequence (cds) comprising of 1713 nucleotides coding for 571 amino acids with the stalk and globular head major areas comprising of 1-143 amino acids and 125-571 amino acids respectively. The transmembrane domains of the selected thermostable were variable with three to five predictable domains at positions 24–47, 25–45, 96–97, 101–107 and 557–563. 13 cysteine (C) residues were conserved for all our isolates at position 123, 172, 186, 196, 238, 247, 251, 344, 455, 461, 465, 531 and 542. Six glycosylation sites were also conserved across all isolates at positions G_1_-119, G_2_-341, G_3_-433, G_4_-481, G_5_-508 and G_6_-538. The details is provided in Table 4. The sequence identity matrix for HN gene of the selected thermostable isolates and other strains of ND-virus are provided in Table 5 and the predicted B-cell epitopes for HN genes are given in Table 6

**Table 4:** Predicted transmembrane amino acid sequences analysis for HN gene of thermostable ND-viruses and vaccine strains

*Potential Transmembrane Domains of thermostable field strains and vaccine strains*					
NDV strain	Start	End	Cutoff	Length	Predicted amino acid sequence
NDV/UG/MU/007 NDV/UG/MU/010 NDV/UG/MU/022 NDV/UG/MU/039 NDV/UG/MU/059 NDV/UG/MU/111	24	47	1.7	24	VFRIAVLSLIIMILVISVSILVYS
	25	46	2.2^a^	22	
	96	97	1.7	2	LL
	101	105	1.7	5	STIMN
	557	563	1.7	7	RIVPLLV
NDV/UG/MU/098	24	47	1.7	24	VFRIAVLSLIIMILVISVSILVYS
	25	46	2.2^a^	22	FRIAVLSLIIMILVISVSILV
	96	97	1.7	2	LL
	101	104	1.7	4	SVIM
	557	563	1.7	7	RIVPLLV
LaSota Thermolabile strain	23	47	1.7	25	IFRIAILFLTVVTLAISVASLLYS
	25	45	2.2^a^	21	FRIAILFLTVVTLAISVASLL
	209	210	1.7	2	GV
	557	563	1.7	7	TTIMN
I-2 Thermostable strain	23	44	1.7	22	VFRIAILFLTVVTLAVSAAALAYS
	25	42	2.2^a^	18	FRIAILFLTVVTLAVSAAALA
	424	429	1.7	6	ALLYPM
	557	563	1.7	7	RIVPLLV
NDV4-C Thermostable	24	44	1.7	21	VFRIAILLSTVVTLAISAAAL
	25	42	2.2^a^	18	FRIAILLSTVVTLAISAA
	210	210	1.7	1	L
	424	430	1.7	7	ALLYMI
	557	563	1.7	7	RIVPLLV
NDV TS09-C thermostable	24	44	1.7	21	VFRIAILLSTVVTLAISAAAL
	25	42	2.2^a^	18	FRIAILLSTVVTLAISAA
	210	211	1.7	2	VL
	424	430	1.7	7	ALLYPMI
	557	563	1.7	7	RIVPLLV
NDV B1 strain	24	47	1.7	24	VFRIAILLSTVVTLAISAAALAYS
	25	45	2.2^a^	21	FRIAILLSTVVTLAISAAALA
	557	563	1.7	7	RIVPLLV

Sequences in boxes show variation in consensus with avirulent strains. The transmembrane domains marked with (^a^) were significant and considered to different isolates and vaccinates

**Table 5:** Sequence identity matrix for CDS of HN gene of thermostable ND-viruses and vaccine strains

SEQUENCE	LASOTAC5	TZ	D58	MU001	MU007	MU010	MU022	MU039	MU059	MU098	MU111	LASOTA	I-2	BEAUDC	C/P/01	I-2PRO	TS09-C	NDV4C	CLONE30
LA SOTA C5	**ID**	**83.2**	**93.4**	**81.1**	**81.2**	**81.2**	**81.2**	**80.9**	**81.2**	**80.9**	**81.1**	**99.5**	**91.3**	**9.6**	**0.8**	**0.8**	**0.8**	**0.9**	**60.7**
TZ060107	76.2	**ID**	**88.0**	**90.8**	**90.7**	**90.5**	**90.7**	**90.5**	**91.0**	**90.8**	**90.8**	**82.8**	**83.2**	**9.4**	**1.5**	**0.7**	**0.7**	**0.8**	**53.0**
D58	93.5	81.3	**ID**	**85.9**	**86.1**	**86.1**	**86.1**	**85.7**	**86.1**	**85.7**	**85.9**	**93.8**	**87.1**	**10.0**	**0.6**	**0.8**	**0.8**	**0.8**	**60.2**
NDV/MU001	76.4	85.8	81.4	**ID**	**98.5**	**98.4**	**99.1**	**99.2**	**98.9**	**98.7**	**99.2**	**80.9**	**80.7**	**8.2**	**1.5**	**0.7**	**0.7**	**0.8**	**51.5**
NDV/MU007	76.7	85.8	81.7	98.4	**ID**	**99.4**	**99.1**	**98.9**	**98.9**	**98.7**	**98.9**	**81.1**	**80.7**	**8.2**	**1.4**	**0.7**	**0.7**	**0.8**	**51.5**
NDV/MU010	76.7	85.8	81.7	98.3	99.8	**ID**	**98.9**	**98.4**	**98.4**	**98.5**	**98.7**	**81.1**	**80.7**	**8.2**	**1.4**	**0.7**	**0.7**	**0.8**	**51.5**
NDV/MU022	76.4	85.8	81.4	99.2	98.8	98.7	**ID**	**99.1**	**99.1**	**98.9**	**99.4**	**81.1**	**80.7**	**8.4**	**1.4**	**0.7**	**0.7**	**0.8**	**51.5**
NDV/MU039	76.4	85.7	81.4	99.3	98.4	98.3	99.1	**ID**	**99.2**	**98.7**	**99.2**	**80.7**	**80.4**	**8.2**	**1.5**	**0.7**	**0.7**	**0.8**	**51.3**
NDV/MU059	76.6	85.9	81.6	99.0	98.5	98.4	99.3	99.0	**ID**	**98.7**	**99.2**	**81.1**	**80.4**	**8.2**	**1.4**	**0.7**	**0.7**	**0.8**	**51.3**
NDV/MU098	76.4	85.8	81.4	99.0	98.6	98.4	99.3	98.9	99.0	**ID**	**99.1**	**80.7**	**80.7**	**8.2**	**1.4**	**0.7**	**0.7**	**0.8**	**51.5**
NDV/MU111	76.6	86.0	81.6	99.1	98.6	98.5	99.4	99.1	99.2	99.2	**ID**	**80.9**	**80.6**	**8.2**	**1.4**	**0.7**	**0.7**	**0.8**	**51.5**
LASOTA	99.7	76.2	93.6	76.3	76.6	76.6	76.3	76.3	76.5	76.3	76.5	**ID**	**90.9**	**9.4**	**0.6**	**0.8**	**0.8**	**0.9**	**60.5**
I-2	87.9	78.6	82.7	77.2	77.3	77.3	77.3	77.1	77.2	77.4	77.3	87.7	**ID**	**10.4**	**1.3**	**0.8**	**0.8**	**0.8**	**56.9**
BEAUDETTE C	9.3	9.3	9.9	9.2	9.3	9.3	9.3	9.2	9.2	9.3	9.2	9.3	10.2	**ID**	**1.5**	**<.001**	**<.001**	**<.001**	**9.5**
C/P/0103/01	5.2	5.7	5.5	5.5	5.5	5.5	5.4	5.5	5.5	5.4	5.5	5.1	5.2	12.9	**ID**	**0.2**	**0.12**	**0.2**	**0.8**
I-2PROGENITOR	3.0	2.8	2.8	2.8	2.8	2.8	2.8	2.8	2.8	2.8	2.8	3.0	3.0	0.2	0.6	**ID**	**89.0**	**89.2**	**0.8**
TS09-C	3.1	2.8	2.9	2.9	2.9	2.9	2.8	2.8	2.9	2.8	2.9	3.1	3.0	0.3	0.5	95.4	**ID**	**99.5**	**0.7**
NDV4-C	3.1	2.8	2.9	2.9	2.9	2.9	2.9	2.9	2.9	2.9	2.9	3.1	3.0	0.3	0.5	95.5	99.8	**ID**	**0.8**
CLONE 30	68.5	55.0	66.8	55.1	55.2	55.2	55.0	55.1	55.1	55.2	55.2	68.5	61.4	9.0	5.0	3.2	3.1	3.1	ID

Amino acid differences are indicated in bold and nucleotide differences are in normal font. The difference with commonly used vaccine strains are high lightened

**Table 6:** B-cell epitopes of HN gene of representative thermostable field ND-viruses

HN GENE B-CELL EPITOPES				
			Thermostable field isolates	NDVUG/MU/059
NO	Start	End	Peptide	Peptide
1	13	18	EEREAK	EEREAK
2	48	58	TGASTPSDLAS	GASTPSDLAS
3	63	69	ISKAEDR	ISKTEDR
4	79	79	E	D
5	115	135	NGAANTSGCGAPVHDPDYIGG	NGAANTSGCGAPVHDPDYIGG
6	146	147	SD	SD
7	150	156	SFYPSAY	SFYPSAY
8	164	174	PAPTTGSGCTR	PAPTTGSGCTR
9	179	180	DM	DM
10	198	201	DHSH	DHSH
11	229	240	LDDTQNRKSCSV	LDDTQNRKSCSV
12	242	244	ATP	ATP
13	255	267	TETEEEDYRSVAP	TETEEEDYRSVAP
14	279	284	GQYHEK	GQYHEK
15	297	307	ANYPGVGGGSL	ANYPGVGGGSL
16	319	331	GLKPNSPSDAAQE	GLKPNSPSDAAQE
17	341	351	NNTCPDEQDYQ	NNTCPDEQDYQARMAKSSYKPGRFGGK
18	357	367	SSYKPGRFGGK	
19	381	395	SLGEDPELTVPPNTV	SLGEDPELTVPPNTV
20	418	420	SSY	SSY
21	436	439	ATLH	ATLH
22	447	461	FTRPGSVPCQASARC	FTRPGSVPCQASARC
23	469	473	VYTDP	VYTDP
24	494	494	N	N
25	496	499	QARL	QARL
26	517	527	VSSSSTKAAYT	VSSSSTKAAYT

*A* Alanine, *R* Arginine, *N* Asparagine, *T* Threonine, *V* Valine, *C* Cysteine, *Q* Glutamine, *E* Glutamate, *D* Aspartate, *G* Glycine, *H* Histidine, *I* Isoleucine, *L* Leucine, *K* Lysine, *M* Methionine, *F* Phenylalaine, *P* Proline, *S* Serine, *W* Tryptophan, *Y* Tyrosine

## Discussions

### Thermostability and infectivity of isolates

Several thermostable strains like I_2_, HR-V4 and V4 have been isolated and characterized [7, 16, 34]. This specific study profiled the thermostability of the Ugandan ND-virus isolates and compared them at HN protein to differentiate local strains from existing vaccines strains. While thermostability is considered to be a natural property of ND-virus isolates used to rapidly characterize field ND-virus isolates in epizootiological studies in the absence of any other marker [13], Ugandan field ND-virus isolates showed disparate thermostability profiles which is not related to the virulence of isolates, site of isolation in the bird species or geographical locality of isolation. This confirms the findings from other studies [20, 33]. Although poultry samples were collected from places of different climatic conditions of their natural sources, thermostability profiles of isolates did not reflect these conditions. The average thermostability of isolates from climatically hotter North and North-East regions of Uganda was 45 min, while that from cooler central and western regions was 60 min. This observation is in agreement with other studies supporting the fact that heat resistance in ND-virus is not a climatically determined trait [15].

The natural spread of clonal derivatives of vaccines strains in a locality following massive vaccination has been observed [3]. In the present study, thermostable isolates were recovered from unvaccinated flocks. This suggests some degree of natural area spread which explains disparate thermostability profiles of both waterfowl and live bird market bird isolates. This is further confirmed by presence of new genotypes our previous study of genotypic characterization of LBM isolates [8]. Taken together, there is a spread of field viruses with varied pathogenicity, thermostability and antigenicity in tropical developing countries, which require a carefully designed control measures.

A prolonged exposure of ND-virus isolates to a high temperature of 56 °C has been preferred to short time exposure at lower temperature during heat selection [17, 41]. This is to ensure selection of a more thermostable ND-virus from a heterogenous population containing a mixture of heat-stable and heat labile strains [19, 22]. We recovered 13.7 % (23/168) of field isolates (LBM and waterfowl) with residual HA titers after 2 h exposure at 56 °C. Of the 23 isolates, 26.2 % (6 out of 23 isolates) had greater heamagglutinin titers greater than reference I_2_ strain. These results are in conformity with other studies especially [19], who reported 38 % of the field ND-virus isolates with a greater heamagglutinin than reference thermostable strains. By regression analysis, we noted thermostable and thermolabile phenotypes of strains that was not influenced by site of isolation. Isolates from oro-pharyngeal (P) and cloaca (C) of the same bird had varied thermostability profiles. This confirms the idea that thermostability is not truly a genotypic character but just a trait that can readily be increased by selection involving heat shock [35].

Like in the Australian experience of enhancing thermostability of V4 vaccine strain from a subpopulation of known heat-stable virus also adopted by [16], we subjected clean ND-virus isolates to the same temperature (56 °C), followed HA titers until 2 h and selected four most thermostable isolates. Previous studies have attempted to isolate avirulent, thermostable ND-viruses from the feces of wild migratory birds for vaccine candidates [32, 33, 40]. Here, the present study identified thermostable ND-viruses from waterfowls and this could provide a promising line of research in thermostable vaccine development in Uganda and providing a base to develop an affordable thermostable ND vaccine.

### DNA sequence analysis of the HN gene

HN is a type II homotetrameric glycoprotein with a monomer length of 577 amino acids for most NDV strains [25]. The heamagglutinin, Neuraminidase and thermostability are one of the multifunctional functions of HN protein that plays key roles in the steps of the NDV life cycle [14]. Mutations within the HN gene has been reported to contribute to thermostability phenotype and immunogenicity of ND-virus [39, 43]. Deletions of amino acid, R 403 were thought to influence thermostability but attempts by several studies to find alterations at such a position was futile in thermostable strains. However, evaluation of chimeric ND-viruses confirmed that thermostability phenotype of ND-virus was dependent upon the origin of HN segment and not any other ND-virus genome [43]. Still no study has offered a clear mechanism or sequence based analysis of HN gene for thermostability. By analyzing the sequences of field thermostable and thermolabile viruses, we noted no deletions at the suggested site but observed increased proportion of charged amino acid residues at the expense of uncharged polar amino acid among the thermostable isolates. This could confer rigidity and stability by minimizing deamination and backbone cleavage.

**Fig. 4:**
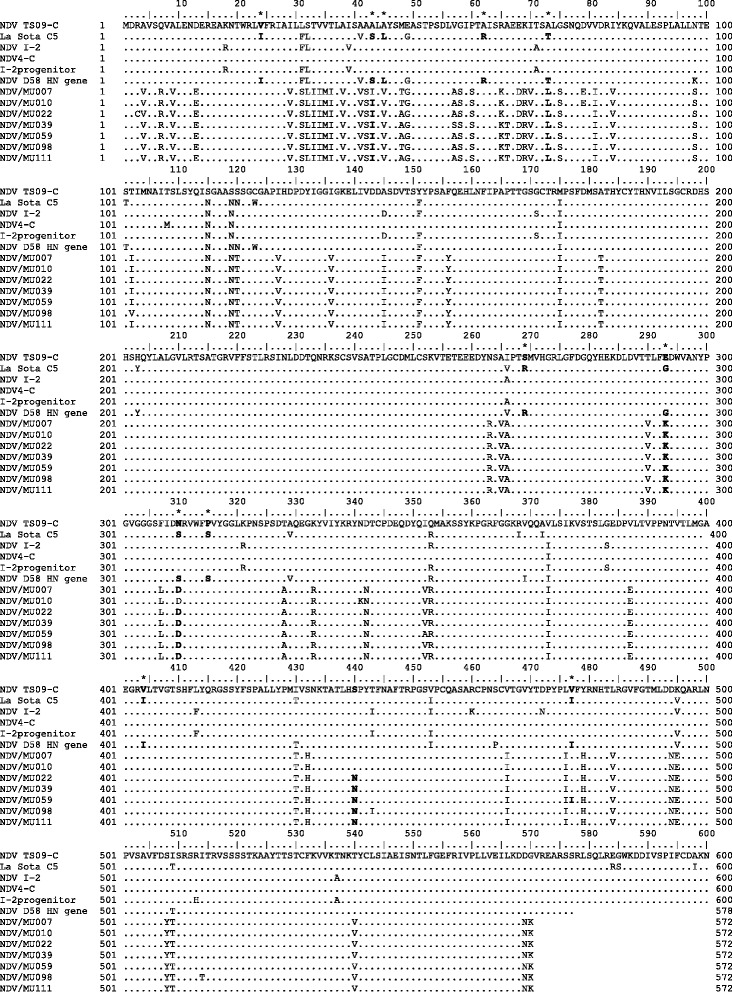
Multiple amino acid sequence alignment of HN proteins. Alignment was performed by dividing amino acids into stabilizing and destabilizing groups. Black bold fonts marked by asterik (*) denote point variation in identity between thermostable and thermolabile viruses

The change to a higher proportion of isoleucine (I), leucine (L), valine (V) and arginine (R) at the stalk and globular regions of NDV HN ectodomain of thermostable isolates offers an explanation to the thermostability since these aliphatic amino acid residues contribute to hydrophobic interaction, a force that maintains the conformational internal protein integrity. Besides, it has been observed that substitution of amino acids at the conserved stalk spike especially the leucine zipper motif affect the Neuraminidase activity of the globular domain where HN molecule interacts with sialic acid binding and F protein to initiate fusion. Protein stabilities have been predicted by sequence feature-based predictions upon amino acid substitutions using models [38]. Our observed amino acids substitutions at V24I, I43S, V45L, T48A, R62A, T73L, V266A, R269S, G293K, S310D, S315P, I404V, I477V and N440S in our thermostable isolates all decreased protein stability with prediction confidence of >80 % which in our case partly explains thermostability phenotype shown in Fig. 4.

Several amino acid residues have been reported as functional amino acid residues, F220, S222, L224, E401, R416, and Y526 in HN protein. 13 cysteine amino acid residues at position 123, 172, 186, 196, 238, 247, 251, 344, 455, 461, 465, 531 and 542. Six potential glycosylation sites at G_1_-119 G_2_^-^341, G3-433, G_4_-481, G_5_508 and G_6_-538, together with the different length of HN cds [29, 31]. The salient features of HN protein of our strains include: 571 amino acid size; conserved amino acids: R 174, I 175, R 197, D 198, K 236, R 416, R 498, Y 526 and E 547; potential glycosylation sites being G_1_-119, G_2_-341, G_3_-433, G_4_-481, G_5_-508 and G_6_-538, which is similar to genotype II vaccine strains Beaudette C, B1 and Ulster. Replacement of glycosylation site G_5_-508 in other ND-virus by tryptophan and not serine (G_5_-N508Y) has been used to pathotype ND-virus as avirulent [29]. All our selected thermostable isolates had this glycosylation site confirming their high pathogenicity reported earlier [8]. Further, a conserved amino acid E347 present in all thermostable and thermolabile isolates, a feature common to all vaccine strains of genotype II ND- viruses was observed. The percent similarity in relation to amino acids between isolates and thermostable I_2_ and TS09C is 80.6 % and that of thermolabile LaSota is 80.5 % suggesting our isolates share some attributes of these vaccine strains shown in Table 5. It could not be establish why isolates obtained from unvaccinated birds share this similarity more so with I_2_ vaccine that has not been widely used in the country at the time of isolation.

The most variable portion of NDV HN protein is present in the N-terminal 78 amino acids that included the transmembrane domain at position 24–47, 25–45, 557–563 using online bioinformatics webserver DAS-transmembrane prediction server [11]. The significant cut off value for the transmembrane domain considered was 2.2 at 25–45 length common to all isolates and vaccinates. However, the variable predicted number of transmembrane domains for thermostable isolates were similar to other thermostable vaccine isolates I_2_, V4 more than those of thermolabile ones. The sialic acid binding sites were all conserved across isolates irrespective of their thermostability phenotypes. This finding is predictable since this site plays a key attachment role of the virus to cells. The presence of cysteine amino acid residue at position 123 has been reported to be essential for intramolecular disulphide bonds that stabilize the oligomeric HN structure gene [10]. Our indicated isolates had conserved cysteine amino acid at position 123 implying absence of chymotrypsin cleavage site and these could form disulphide-linked dimers increasing the hydrophobic properties of the entire HN molecule responsible for thermostability after exposure to 56 °C. The R416 essential for receptor binding, and Neuraminidase (NA) and haemagglutination (HA) activity was likewise conserved even after exposure to 56^o^ C, as were the receptor binding site involving E401, R416 and Y526 [9]. The predicted amino acids present in the transmembrane domain of our isolates ^25^FRIAVLSLIIMILVISVSILVY^46^ which is different from less virulent thermostable I_2_ or TS09C (Table 6). This variation could be due the virulence difference of these two categories.

The B-cell epitopes of thermostable isolates were 26 except for one isolate NDV/UG/MU/059 having 25 epitopes with four and 24 epitopes in the stalk and globular regions respectively (Table 6). This finding is consistent with earlier finding of the immunodorminant epitope concentrated in the globular region [10]. Earlier studies have confirmed that immune system responds to HN protein rather than the F gene and confirmed by use of neutralizing monoclonal antibodies with overlapping site at the HN gene. This variations of our isolates by HN gene suggests the existence of thermostable strains different from vaccine strains as already confirmed by our earlier findings using the HN phylogenetic tree analysis constructed by neighbor joining (NJ) algorithm with bootsrap along with distance.

## Conclusion

From the current study, it can be concluded that field ND-virus (virulent or avirulent) circulating in LBM and waterfowl in Uganda display disparate thermostability profiles. Waterfowl provides a better source of avirulent, thermostable isolates with more than 4 logarithmic orders of HA within one hour of incubation at 56 °C and without loss of two logarithmic orders of infectivity titer within 30 min at serial passages. Chimeric NDV molecules has yielded new knowledge towards the understanding of NDV thermostability these require obtaining metastable HN molecules and performing mechanistic studies on how thermostability phenotype can be predicted by analysis of HN gene and such changes in amino acids of HN gene might contribute to the stability of the ND-virus strain at high temperature.

### Accession numbers

The complete HN gene sequences of isolates analyzed in this study are deposited in GenBank with accession numbers; Isolates [HG937536, HG937538, HG937542, HG937548, HG937553, HG937564 and HG937566], vaccinates [JX110635, AY935499, AY935500, JX443519, JF950510, KC844235 and EU305607].

## Abbreviations

EID_50 %_: Median Embryo Infectivity Dose
HA: Hemagglutination test.
ND-virus: Newcastle disease virus
PBS: Phosphate buffered saline
RBCs: Red blood cells
